# ACEs Aware: Translating Screening for Adverse Childhood Events and Trauma-Informed Care to Address the US Noncommunicable Disease Epidemic

**DOI:** 10.5888/pcd23.260486

**Published:** 2026-11-12

**Authors:** Andrés Arana, Conqualla Scott, Richard Cristian Brito

**Affiliations:** 1 Department of Population Health, Grossman School of Medicine, New York University, New York, New York; 2 Department of Population, Family, and Reproductive Health, Johns Hopkins Bloomberg School of Public Health, Baltimore, Maryland; 3 Institute of Public Health, College of Pharmacy and Pharmaceutical Sciences, Florida Agricultural and Mechanical University, Tallahassee, Florida

Noncommunicable diseases (NCDs), including cardiometabolic diseases, diabetes, mental illness, and cancer, account for more than 70% of deaths annually in the US (1). NCDs are chronic illnesses caused by exposome interactions, encompassing genetics, physiology, behavior, and the environmental factors that cannot be transmitted between people (1). For more than a century, NCDs have substantially limited the health and well-being of the US population, contributing to premature death for people who have NCD comorbidities (1).

Although most NCDs are preventable, multiple modifiable risk factors, including diet, physical activity, and substance use, directly influence their occurrence and progression (1). Research studying the root causes of chronic illnesses, including the developmental origins of health and disease (DOHaD) and life course health development (LHD) frameworks, demonstrated an increased risk of all-cause NCD onset, comorbidity, and mortality among adults who experienced early life adversity (2,3). Unfavorable environments during critical developmental periods before adulthood also increase the susceptibility of engaging in unhealthy behaviors (eg, substance use or misuse as learned negative coping strategies through childhood exposure to a caregiver’s behaviors) while managing chronic illnesses during adulthood (4–6). This dynamic presents additional barriers to care and disease management (4).

Since the foundational 1998 study by Felitti and colleagues, a body of literature investigating the long-term effects of adverse childhood experiences (ACEs) has shown that enduring abuse, neglect, or household dysfunction before the age of 18 years significantly increases individual risk of an NCD prognosis (1,5). ACEs are commonly assessed through a validated 10-item questionnaire, in which a dose–response effect reflects the cumulative risk that ACEs pose to health outcomes. Studies indicate that people scoring 4 or higher on the ACEs questionnaire face an exponentially higher risk of being diagnosed with an NCD during early adulthood (1–6). Additionally, this negative association is further supported by DOHaD and LHD, showing multiple exposome pathways through which ACEs predispose people to NCDs. These pathways include biological and epigenetic modifications associated with elevated cardiometabolic risk, psychological mechanisms increasing the severity and duration of toxic stress, and various psychopathologies over the life course (5–8).

ACEs and NCDs disproportionately affect people living with poor social determinants of health (SDoH) (5,6,9). These systemic inequities deteriorate the quality and access of available resources in their residing environment, negatively affecting the health and well-being of vulnerable communities, including people with low socioeconomic status and historically underserved populations (5,6,9).

The DOHaD, LHD, and SDoH frameworks provide a comprehensive patient-centered health care delivery model focused on how early life experiences, as well as the exposome, influence health outcomes across the lifespan (2,3,6,9). Moreover, NCD management among populations potentially exposed to ACEs demands an evidence-based, trauma-informed care approach to provide a safe space and to prevent retriggering, retraumatizing, and stigmatizing patients in clinical practices (9). For instance, a 2024 study by Machtinger et al found that incorporating a trauma-informed care approach significantly increased the rate of screenings for ACEs, showing improved patient engagement, which can aid in preventing the long-term effects of ACEs on NCDs (9,10).

Although a direct correlation exists between ACEs and NCDs, their clinical assessment and potential health implications are not integrated into most US health care settings (8–10). Without this information, providers and health systems lack a critical, validated tool that identifies at-risk patients whose treatments would substantially benefit from patient-centered practices accompanying their care (9,10). Consequently, integrating trauma-informed care and ACEs screening to acknowledge the origins of these NCDs and how they could alter common medical interventions is pivotal in fostering positive, patient-centered health trajectories.

## Case Study: Medi-Cal ACEs Aware Initiative

Embedding ACEs screenings into patient records helps optimize the provision of state and local resources in health care systems (9,10). As such, a collaboration between the California Department of Health Care Services and the Office of the California Surgeon General launched the *ACEs Aware* initiative in 2019, becoming the first state-level effort in the US to promote early ACEs detection and intervention in primary care practices (10). *ACEs Aware* recognizes that relying solely on pharmacological interventions in primary care is insufficient to address the needs of patients with NCDs. Accordingly, their practice comprehensively integrates SDoH, enhancing patient reach and access to quality health care, food, workforce, and education, all of which directly influence their daily predisposition and presence of trauma and toxic stress outside of the clinic (10).

*ACEs Aware* incentivizes California’s Medicaid safety-net provider network, Medi-Cal, to conduct ACEs screenings by reimbursing them following the fee-for-service model (10). This system directly connects people who reported at least 1 ACE with patient-centered treatments personalized to their disease and risk profile. Moreover, Medi-Cal serves nearly 14 million people in the state, most of whom have low incomes and are children; thus, approximately one-third of California’s population is eligible for screenings (10). Moreover, *ACEs Aware* shows that structured trauma-informed care networks can be matched with financial reimbursements for community partners to systematically improve the SDoH where patients reside (9,10). For example, medical practitioners are trained to screen for signs of toxic stress using the *Pediatric ACEs Screening and Related Life Events Screener* (*PEARLS*) tool and to enter the corresponding billing code directly into the patient’s electronic health record (10). During a routine visit, providers administer the PEARLS screening, assess risk and protective factors, and refer them to the appropriate community network social workers to help connect caregivers with available services and resources to address the deficiencies set by their SDoH (9,10).

Making the initiative available to the public provides an equitable, accessible blueprint that could be adapted and implemented into other health systems (10). Bridging the gap between early-life experiences and clinical care using an *ACEs Aware* model is a feasible strategy to improve treatment effectiveness, reduce health inequities, and lower NCD prevalence in the US (7,9,10).

## Limitations and Future Directions

Although the consensus from public health advocates supports ACEs screenings, several limitations in research, policy, and practice have hampered implementation efforts (7,9,10). For instance, most clinical settings attempting to conduct ACEs screenings lack structural pathways to readily and sustainably refer patients to adequate resources. Moreover, arguments about the risk for patients, including stigma and retraumatization, may discourage providers from conducting the screening (9). California’s systematic implementation of ACEs screenings serves as an example showing how to overcome these barriers by leveraging existing resources and establishing a centralized hub, incentives, competent training for trauma-informed care, and a multidisciplinary network to integrate screenings into routine care (10). *ACEs Aware* follows a trauma-informed care approach, improving the health care experience for patients and providers by creating a safe environment where they can discuss the factors influencing disease management, making the implementation of ACEs screening an effective component shaping health care delivery (9).

*ACEs Aware* presents a scalable, evidence-based initiative that can be feasibly incorporated into existing US health care systems (10). However, these efforts should be guided by implementation science to ensure an adequate adaptation of the initiative to meet specific and multilevel needs across diverse settings (7,10). Additionally, not all patients are eligible to participate in this initiative because some populations do not qualify to receive Medi-Cal services, which leaves most of the population ineligible to receive screenings through a federally funded initiative, thus limiting the adoption of nationwide ACEs screenings (9).

## Conclusion

Health care systems have historically been underprepared for addressing challenges to disease management, especially when primarily relying on complex, downstream lifestyle changes without accounting for relevant contextual information (9). To effectively address the persisting NCD epidemic that contributes to the national mortality rate, initiatives such as *ACEs Aware* can be an exceptional adaptation of the provision of health care. Across primary health care settings, ACEs screening could serve as a diagnostic pathway, allowing health systems to proactively address their potential negative influences by providing trauma-informed care. Additional research evaluating how *ACEs Aware* improved specific NCD metrics for different populations, such as hypertension and obesity among vulnerable people, could help public health partners prioritize their efforts to better serve community-specific needs. Moreover, expanding *ACEs Aware* to include all participants regardless of Medi-Cal eligibility status would benefit population health outcomes. This initiative offers an innovative approach to advancing trauma-informed care by consolidating its fundamental multifaceted tenets and translating them into actionable practices, ultimately providing practitioners with clear protocols to connect patients with a history of ACEs with available services and resources to address the NCD epidemic.
